# Fostemsavir resistance-associated polymorphisms in HIV-1 subtype C in a large cohort of treatment-naïve and treatment-experienced individuals in Botswana

**DOI:** 10.1128/spectrum.01251-23

**Published:** 2023-10-12

**Authors:** Boitumelo J. L. Zuze, Botshelo T. Radibe, Wonderful T. Choga, Ontlametse T. Bareng, Natasha O. Moraka, Dorcas Maruapula, Kedumetse Seru, Patrick Mokgethi, Baitshepi Mokaleng, Nokuthula Ndlovu, Nametso Kelentse, Molly Pretorius-Holme, Roger Shapiro, Shahin Lockman, Joseph Makhema, Vlad Novitsky, Kaelo K. Seatla, Sikhulile Moyo, Simani Gaseitsiwe

**Affiliations:** 1 Botswana Harvard AIDS Institute Partnership, Gaborone, Botswana; 2 Department of Medical Sciences, Faculty of Allied Health Professions, University of Botswana, Gaborone, Botswana; 3 Department of Biological Sciences, Faculty of Science, University of Botswana, Gaborone, Botswana; 4 Department of Immunology and Infectious Diseases, Harvard T.H. Chan School of Public Health, Boston, Massachusetts, USA; 5 Brigham and Women’s Hospital, Boston, Massachusetts, USA; National Institute of Allergy and Infectious Diseases, Baltimore, Maryland, USA

**Keywords:** HIV-1 C, entry inhibitors, fostemsavir (FTR), drug-resistant mutations (DRMs), polymorphisms, Botswana

## Abstract

There are limited data on the prevalence of fostemsavir (FTR) resistance-associated polymorphisms in people with HIV in Botswana. We sought to determine the prevalence of FTR resistance-associated polymorphisms in HIV-1 subtype C sequences from antiretroviral therapy (ART)-naïve and ART-experienced individuals in Botswana. Previously reported FTR resistance-associated polymorphisms from literature were surveyed from HIV-1C proviral sequences generated from a large HIV-1 population-based cohort enrolled between 2013 and 2018. Sequences from ART-naïve and ART-experienced individuals were included in the analysis. A total of 6,078 participants with gp120 sequences were included in this study, and 6,030 (99.2%) had known ART status; 1,282 (21.3%) were from ART-naïve individuals, while 4,748 (78.7%) were from individuals on ART at study enrollment. Viral load (VL) data were available for 4,739 (99.8%) ART-experienced individuals, of whom 4,526 (95.5%) were suppressed and 213 (4.5%) had virologic failure (VF) (VL >400). Among those with VF and ART naive, the overall prevalence of FTR resistance was 13.3% (95% CI 11.6%–15.1%) and did not differ between ART-experienced with VF individuals (29/213, 13.6%) and ART-naïve individuals (170/1,282, 13.3%) (*P*-value = 0.9). The most predominant mutations (prevalence ≥1.0%) were M434I (9.8%), M475I (5.9%), and M426L (1.1%). The overall prevalence of FTR polymorphisms was similar in both ART-naïve and ART-experienced individuals with VF in a setting with no prior FTR exposure. We recommend further studies on the phenotypic impact of these polymorphisms to guide how to monitor for FTR resistance. (This study has been registered at ClinicalTrials.gov under registration no. NCT01965470.)

**IMPORTANCE:**

Fostemsavir (FTR) is a newly licensed antiretroviral drug that has been shown to have activity against HIV-1. The mechanism of action of FTR is different from all currently available antiretrovirals (ARVs), and as such, it offers hope for HIV-1 suppression in those people with HIV (PWH) who harbor HIV-1 variants with drug resistance mutations to currently used ARVs. Using 6,030 HIV-1 sequences covering the HIV-1 envelope from PWH in Botswana who are antiretroviral therapy (ART) naïve as well as those who are failing ART, we explored the sequences for FTR resistance-associated polymorphisms. We found the prevalence of FTR polymorphisms to be similar in both ART-naïve and ART-experienced individuals with VF in this setting, with no prior FTR exposure. Further studies on the phenotypic impact of these polymorphisms are warranted to guide how to monitor for FTR resistance.

## INTRODUCTION

The burden of human immunodeficiency virus (HIV) is still a public health concern especially in sub-Saharan Africa (SSA), which shoulders close to two-thirds of the global HIV cases (1, 2). Botswana has a high HIV prevalence rate of about 20.8% but is also the third country globally to reach the Joint United Nations Programme on HIV/AIDS (UNAIDS) 95-95-95 goals (3). Despite this success, drug resistance mutations (DRMs) pose a threat to the efficiency of antiretroviral therapy (ART) in countries such as Botswana where there is high ART coverage (3 – 7). Reports of individuals harboring HIV-1 variants with resistance to most of the available antiretrovirals (ARVs) have been made in Botswana and other countries in SSA (8 – 10). There is, therefore, a need to evaluate the prevalence of polymorphisms to new ARVs such as fostemsavir (11) [approved by the Food and Drug Administration (FDA) and European Medicines Agency] that offer an alternative to people with HIV with limited treatment options (12 – 14).

FTR is a gp120 attachment inhibitor that prevents HIV-1 from interacting with host cell CD4 receptors with subsequent inhibition of virus binding, fusion, and entry into host cells (15). It acts as a conformational blocker at low concentrations, preventing the sampling of downstream conformations required for viral entry (16, 17). According to *in vitro* studies, FTR binds to the HIV-1 gp120 under the anti-parallel β20-β21 sheet and adjoining to the CD4-binding loop (16, 18, 19), stabilizing the “closed” conformation that is ineffective at engaging the CD4+ receptor (16).

The mechanism of inhibition of FTR differs from other entry inhibitors targeting co-receptor binding (maraviroc) or fusion (enfuvirtide) (16). *In vitro* data demonstrate it is active against CCR5-, CXCR4-, and dual-tropic viruses and against almost all HIV-1 subtypes tested, except for subtype CRF01_AE (AE) and possibly HIV-1 group O (20). FTR is a prodrug of temsavir, and studies show no cross-resistance between it and other antiretrovirals (21, 22); therefore, previous regimens should not affect the efficacy of FTR. Similarly, the two-cohort phase 3 randomized placebo-controlled double-blind clinical trial called the “BRIGHTE” study looked at virologic (genotypic and phenotypic) outcomes of heavily treatment-experienced individuals from 22 countries across Africa, Asia-Pacific, Europe, North America, and South America (23). This study evaluated protocol-defined virologic failure (VF) in participants with multi-drug resistance (MDR) HIV-1 FTR optimized background therapy (OBT), the secondary efficacy outcome measure at Week 96 of open-label fostemsavir plus OBT in the randomized cohort using the FDA snapshot algorithm, was the number of participants achieving HIV-1 RNA <40 c/mL (ClinicalTrials.gov Identifier: NCT02362503). These individuals showed sustained virologic suppression through 96 weeks (60% in the randomized cohort participants, 37% in the non-randomized cohort participants) (23).

However, it is important to evaluate for FTR resistance-associated polymorphisms in HIV-1 sequences from a population with high HIV prevalence dominated by HIV-1C and a mature ART program. Here, we sought to assess the baseline prevalence of FTR resistance-associated polymorphisms among HIV sequences from treatment-naïve and -experienced PWH in Botswana. The findings of this analysis will guide the use of FTR as part of a rescue ART regimen for heavily treated PWH who harbor HIV-1 variants with MDR mutations.

## MATERIALS AND METHODS

### Study design and population

This is a retrospective cross-sectional study utilizing already generated 6,078 HIV-1C sequences covering the entire gp120. The generated sequences were from PWH aged 16–64 years residing in 30 communities in northern, central, and southern parts of Botswana who were randomly selected to participate in the Botswana Combination Prevention Project (BCPP) (4, 24 – 26). BCPP enrolled both PWH and HIV-negative individuals. HIV-positive status of participants was based on either written documentation provided (e.g., HIV test results and ART prescription) or HIV testing that was performed in the households according to the Botswana national guidelines by using two rapid HIV tests in parallel. Most of the ART-experienced PWH were on efavirenz- or nevirapine-based ART which was the first-line regimen prior to dolutegravir-based first-line ART implementation in June 2016.

### Near full-length HIV genotyping

Viral sequences were generated by a long-range HIV genotyping protocol described elsewhere (24, 27) with minor modifications that included a reduced annealing temperature (58 °C instead of 62 °C) as a backup amplification strategy and using the first-round amplicon as a template for next-generation sequencing (NGS). Proviral DNA templates were used for amplification, as the majority of participants were receiving ART. The NGS was performed by the Biopolimers Facility at Harvard Medical School (https://genome.med.harvard.edu/) and through collaboration with PANGEA HIV consortium (24) at the Wellcome Trust Sanger Institute (Cambridge, UK; http://www.sanger.ac.uk/) with high-sequencing coverage and using Illumina platform MiSeq and HiSeq. A single consensus sequence represented the population of viral quasispecies per participant.

### Selection of study participants

**Fig 1:**
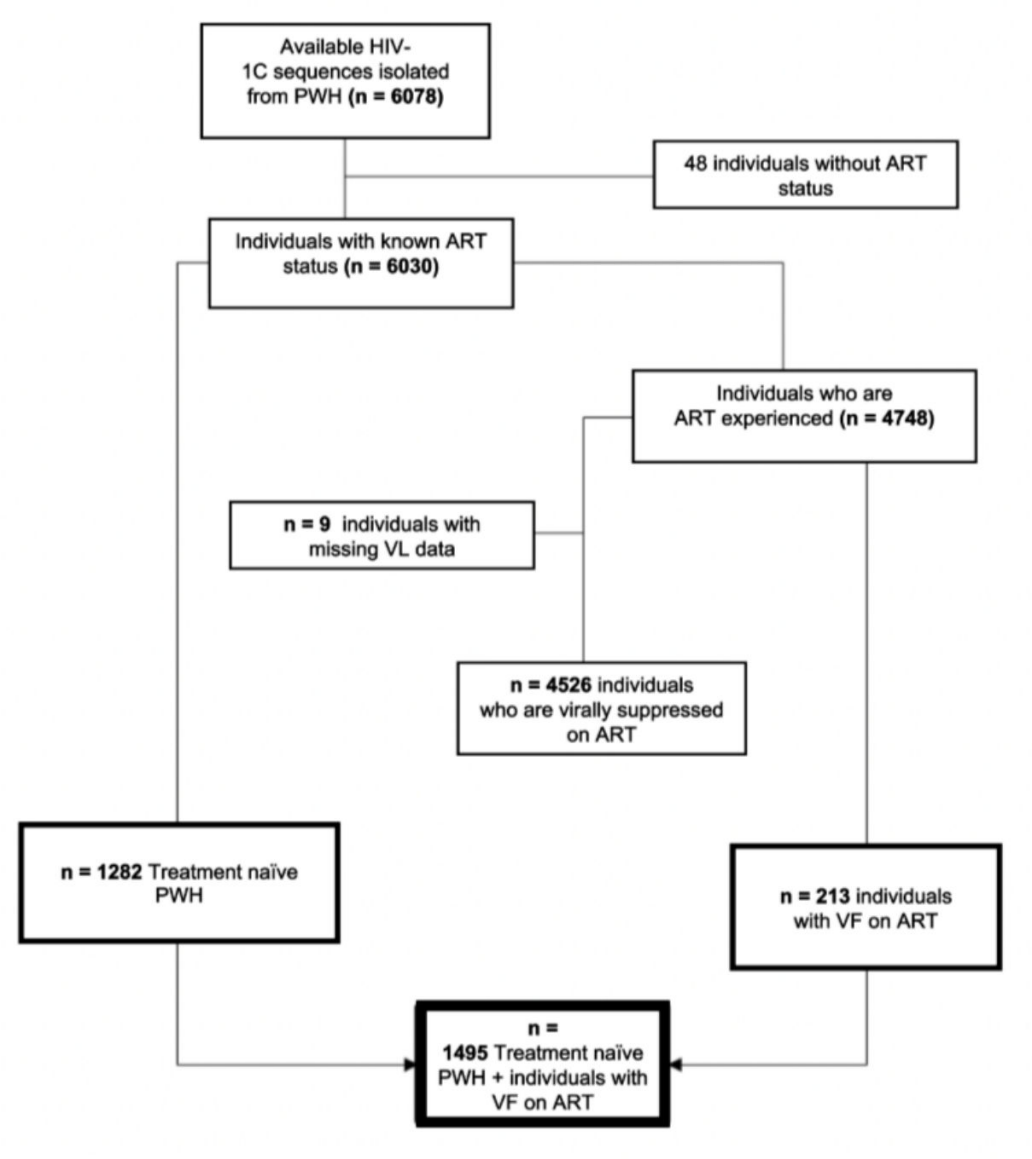
Study schema for specimen selection. *n*, population size.

Only PWH who were either ART naïve or ART experienced with HIV-1 viral load (VL) measurement at the first BCPP study visit and had available HIV-1 gp120 sequence were included in the current analysis. HIV-1 VL of participants was quantified using Abbott m2000sp/rt assay (Wiesbaden, Germany) with a range of 40–10^7^ copies/mL (24). ART-experienced individuals were further sub-divided into virally suppressed individuals and those with VF. Any VL measurements ≤400 copies/mL were considered as viral suppression, while VF was defined as VL >400 copies/mL as per Botswana ARV treatment guidelines (24). Figure 1 is a schema used to perform data sorting and show a proportion of sequences with missing clinical data and was not included in the current analyses.

### Mutation analysis

A total of 6,078 complete HIV-1C sequences were available for analysis. We used Gene cutter tool (https://www.hiv.lanl.gov/content/sequence/GENE_CUTTER/cutter.html) to extract the gp120 fragment of the HIV envelope gene. The obtained nucleotides were aligned using NextAlign (28) and HXB2 as HIV reference sequence. The subsequent multiple sequence alignment (MSA) was visually inspected in Aliview before translating to the corresponding amino acids which was then used for sequence analysis. We developed an in-house python script to curate all the mutations within the MSA. The wild-type mutations were based on the HXB2. Consequently, the functionally characterized FTR resistance-associated polymorphisms were curated from different sources and were used to determine the prevalence of FTR polymorphisms in the present study (Table 1).

**TABLE 1:** FTR resistance-associated polymorphisms and their mode of action

Drug	Mode of action	Drug resistance mutations		Envelope region	Associated drug resistance mechanisms	References
FTR	Binds to the HIV-1 gp120 under the anti-parallel β20-β21 sheet and adjacent to the CD4-binding loop, preventing the viral connection to the CD4	S375	M	β16	Leads to a loss of van der Waals and potential hydrogen-bonding interactions, physically hindering attachment inhibitors from binding	(29)
			H		Results in the spontaneous sampling of an Env conformation closer to the CD4-bound state	(15)
			T Y		Increases sCD4 sensitivity and also induces a distinct trimer conformation	(30)
		S375I + M426L		β16 and β20	Confer high-level resistance of anti-entry activity	(31)
		A281V		ℒC	It increases resistance to neutralization	(32)
		M475 + M426L		α5 and β20	Decreases the size of the attachment inhibitor binding site	(15)
		L116Q		α1	Reduces T-cell binding affinity in HLA-B (major histocompatibility complex, class I, B)	(18)
		M434		β21	May affect trimmer formation and exposes neutralizing antibody epitopes	(33)

### Statistical analysis

The baseline demographics were summarized using descriptive statistics. Categorical variables were reported in percentages while continuous variables in medians with interquartile ranges. Wilcoxon rank-sum test was used to compare continuous variables (VL, age) and chi-square test for categorical variables (district and gender) among these two groups: (i) ART naïve and (ii) virologic failure on ART.

The prevalence of polymorphisms among different groups was estimated with 95% confidence intervals using binomial exact method. The prevalence of polymorphisms was compared among groups using comparison of proportion test. All the statistical analyses were conducted using STATA version 14 software (Stata Corp LP, College Station, Texas, USA), and *P*-value ≤0.05 were considered statistically significant.

## RESULTS

### Participant characteristics

A total of 6,078 HIV-1 near full length sequences were generated for the BCPP study. Of the 6,078 participants with HIV-1 sequences, 48 participants had no ART status and were thus left out from the analysis. Of the remaining 6,030 participants, 1,282 participants were ART naïve and 4,748 were ART experienced. The ART-experienced participants were further stratified to ART experienced and virally suppressed (VL ≤400 copies/mL) [4,526 (95.5%)] and ART experienced with VF (detectable VL >400 copies/mL at entry) [213 (4.5%)], and nine participants had missing VL data. The analysis here focused on those participants who were ART naïve and those who were ART experienced with VL, giving a total of 1,495 participants. The median age at enrollment was 34 years (Q1, Q3: 27.0, 42.0), 70.8% were female, and the median log10 viral load was 4.23 (Q1, Q3: 3.45, 4.80), and these characteristics had no significant difference in the ART naïve and ART experienced with VF. The district category showed statistical significance with the central district having the highest number of individuals.(Table 2)

**TABLE 2:** Baseline demographics for included participants^*a*^

Characteristics	Total (*n* = 1,495) (%)	ART naïve (*n* = 1,282) (%)	On ART with virologic failure (*n* = 213) (%)	*P* values
District				0.01
Northern	269 (18.0%)	216 (16.8%)	53 (24.9%)	
Southern	574 (38.4%)	504 (39.3%)	70 (32.9%)	
Central	652 (43.6%)	562 (43.8%)	90 (42.2%)	
Gender				0.6
Male	501 (29.2%)	426 (33.2%)	75 (35.2%)	
Female	994 (70.8%)	856 (66.8%)	138 (64.8%)	
Median age in years				
(*Q*_1_ *,Q*_3_)	34.0 (27.0, 42.0)	34.0 (27.2, 42.0)	34 (26.7, 41.0)	0.6^*b*^
Median				
log_10_ (viral load)(*Q*_1_ *,Q*_3_)	4.23 (3.45, 4.80)	4.26 (3.53, 4.82)	4.03 (3.21, 4.71)	0.05^*b*^

^*a*^ *n* = sample size; Q1, first quartile; Q3, third quartile.

^*b*^ *P*-values obtained using Wilcoxon rank-sum test while other *P*-values were from chi-square test. Numbers in brackets are percentages within columns.

### Overall prevalence of FTR resistance-associated polymorphisms among ART naïve and individuals experiencing VF on ART

**Fig 2:**
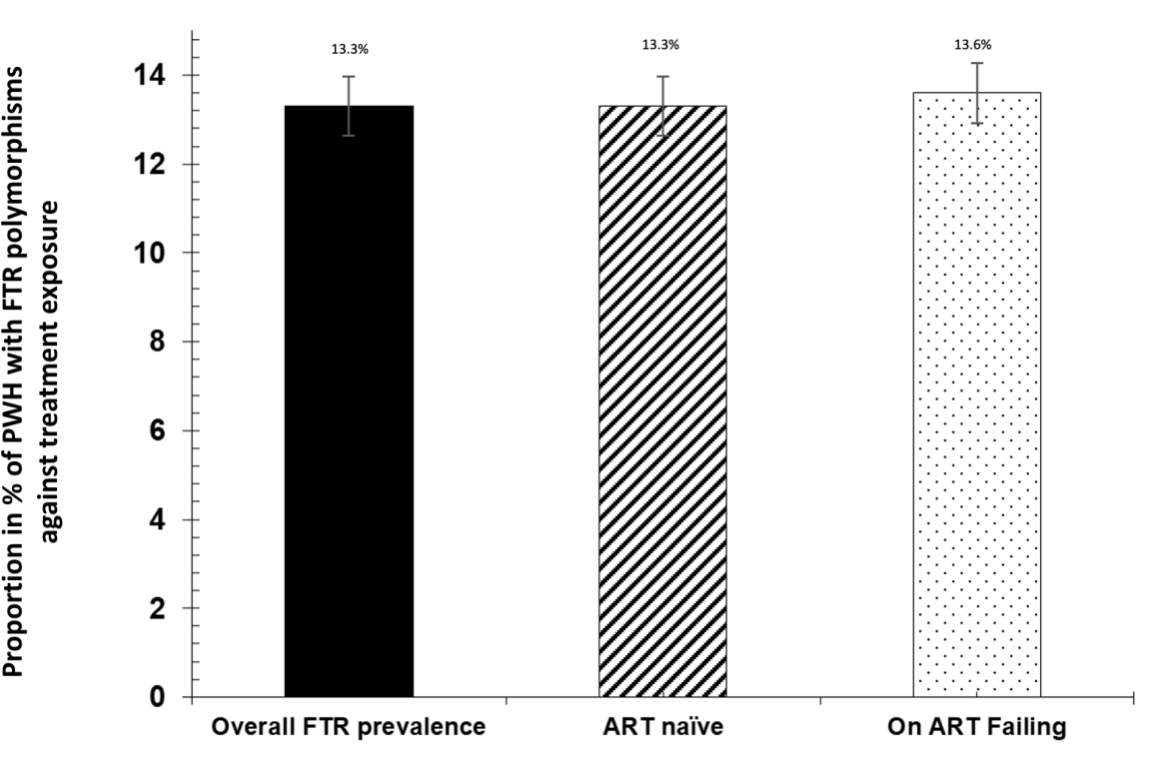
Overall prevalence of individuals with FTR resistanceassociated polymorphisms among ART naïve and individuals experiencing VF on ART. The analysis focused on ART naïve (*n* = 1,282) and on ART with VF (*n* = 213) individuals, total 1,495 individuals, only 199 had FTR polymorphisms.

A total of 1,495 participants were analyzed for FTR resistance-associated polymorphisms. Of these, 1,282 (85.8%) were ART naïve, while 213 (14.2%) were individuals experiencing VF on ART with HIV-1 VL >400 copies/mL. The overall prevalence of participants with HIV-1 harboring FTR resistance-associated polymorphisms was 199/1,495 [13.3% (95% CI: 11.6–15.1)]. By ART status, the prevalence of FTR resistance-associated polymorphisms was 171/1,282 [13.3% (95% CI: 11.5–15.3)] among ART-naïve individuals and 29/219 [13.6% (95% CI: 9.6–18.9)] in those experiencing VF on ART, with no statistically significant difference (Fig. 2).

### Overall prevalence of specific FTR resistance-associated polymorphisms in ART naïve and individuals experiencing VF on ART

**Fig 3:**
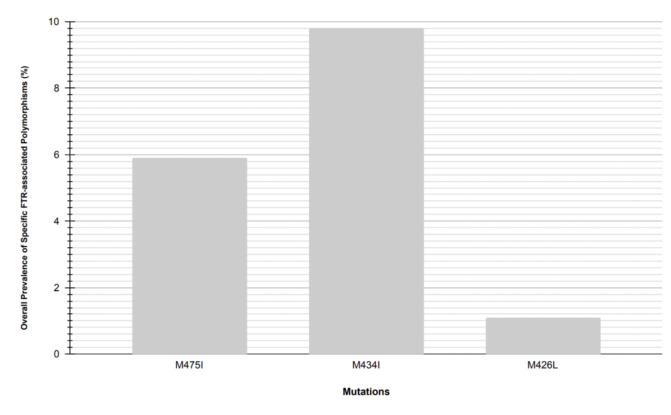
Overall prevalence of specific FTR resistance-associated polymorphisms (≥ 1.0%). Of the 32 detected amino acid substitutions, only 3 had a prevalence (greater than or equal to) 1.0%, these were M475I, M434I, and M426L.

**Fig 4:**
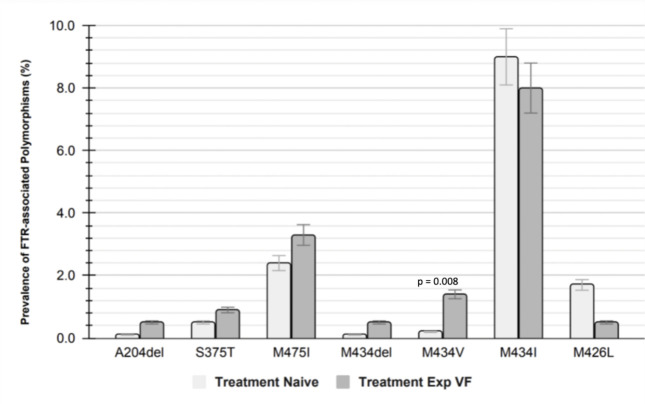
Prevalence of specific FTR resistance-associated polymorphisms among ART naïve and ART experienced with VF individuals. Of the 32 detected amino acid substitutions, 7 were found in both ART naïve and on ART with VF, these were A204del, S375T, M475I, M434del, M434V, M423I, and M426L, having only M434V being statistically significant between these groups (*P*-value <0.05%).

A total of 32 mutations (all shown in Table S1) were found in 979 individuals (16.3%), of which three had prevalence >1.0%: M475I (5.9%), M434I (9.8%), and M426L (1.1%) in Fig. 3. Among all these mutations, M434I was the most frequent polymorphism. When comparing the FTR resistance-associated polymorphisms between treatment naive and treatment experienced with VF, only seven were found: A204del, S375T, M475I, M434del, M434V, M434I, and M426L. Only M434V was statistically significantly higher among individuals with VF on ART (1.4%) compared to 0.2% among ART naïve (*P*-value = 0.008%) (Fig. 4). Additionally, we predicted the tropism of the HIV-1 sequences and observed that R5 tropism was the most dominant viral tropism with 94.8% among individuals with FTR resistance-associated polymorphisms, the remaining 5.2% were CXCR4 tropic and were reported among individuals with VF on ART.

## DISCUSSION

Our study evaluated the prevalence of pre-existing FTR resistance-associated polymorphism in HIV-1 variants from ART-naïve and ART-experienced PWH with VF in Botswana. We report a similar prevalence of FTR resistance-associated polymorphism in HIV-1C from both ART naïve and those on ART with VF. Our study is the first to evaluate FTR resistance-associated polymorphisms among PWH in Botswana and provides guidance on the potential use of this new drug.

**Fig 5:**
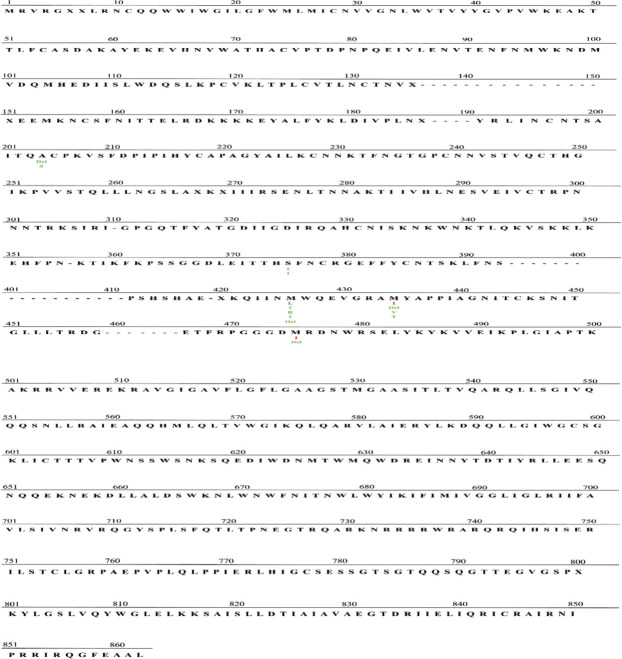
HIV-1 envelope group C amino acid profile. HIV-1 envelope group C amino acid profile. The consensus sequence is indicated in black. Non-consensus amino acids with prevalence (greater than or equal to) 5.0%, 1.1%–4.9%, and 0.1%–1.0% are indicated in red, blue, and green, respectively.

The overall prevalence of FTR polymorphism in this cohort was 13.3%. Our findings are generally consistent with other data regarding FTR resistance, with a few differences. The most prevalent polymorphism in our study was M475I and M434I (prevalence >5.0%), and we observed lower frequency of polymorphisms for M426L (1.1%) and S375T (0.4%) (< 5.0%)(Fig. 5). In contrast, other studies have reported S375T as the most frequent amino acid substitution associated with FTR [13.21% in the different subtypes that were tested (B and non-B) (34), 17.73% in subtype B (18)]. Amino acid substitution M426L was the second most frequent in other studies with 7.56% in subtype B (18) and 6.8% in overall of different subtypes, higher in subtype B (7.9%) than in non-B subtype (4.8%) (34), these compared to the 1.1% found in this study. Amino acid substitutions M434I (9.8%) and M475I (5.9%) were the most predominant in the assessed sequences, this prevalence was higher than most reported in other subtypes [positions 375, 464, 434, and 475 all above 1% (18, 34 – 36)]. This also shows that these substitutions at positions 434 and 475 are not HIV-1 subtype specific (34, 37, 38). According to Nowicka-Sans et al., based on *in vitro* studies, these substitutions have a high resistance to temsavir (GSK2616713, formerly BMS-626529) of which FTR is its prodrug (21). To date, all these substitutions (M426L, S375T, M475I, and M434I) are known to reduce FTR susceptibility both *in vitro* and *in vivo* (16, 21, 35, 39, 40).

We additionally looked into the group of individuals who were treatment experienced and virally suppressed shown in Table S1. Their overall prevalence of FTR resistance-associated polymorphisms 780/4,526 [17.2% (95% CI: 16.1–18.3)] and when comparing to the 171/1,282 [13.3% (95% CI: 11.5–15.3)] among ART-naïve individuals and the 29/219 [13.6% (95% CI: 9.6–18.9)] in those experiencing VF on ART, there was no statistically significant difference. Though when looking at the polymorphisms closely across the ART statuses, amino acid substitutions M475I and M434V were significantly higher in the individuals who were virally suppressed on ART than those with VF on ART.

The study had a good representative sample of PWH in Botswana with a large data set, which revealed a low prevalence of FTR-associated resistance. However, this study had several limitations. It was difficult to pinpoint how each polymorphism affects the efficacy of FTR in HIV-1C, as none of the individuals had any exposure to FTR prior to this analysis. Currently, there is no software/database that is used to predict the effect of FTR mutations, and the mutations reported were based on the available literature. We, therefore, cannot conclude on how many individuals are susceptible or have intermediate and high-level resistance to FTR, although we provide a population-level indication of patterns of polymorphisms in FTR-naive individuals in Botswana, which provides a baseline prevalence of possible polymorphisms associated with FTR- associated resistance.

The importance of this study is that despite Botswana and other countries reaching the UNAIDS 95-95-95 goals (3) and having high ART coverage, drug resistance mutations pose a threat to the efficiency of ART (3 – 7). There are reports of individuals harboring HIV-1 variants with resistance to most of the available ARVs in Botswana and other countries in SSA (8 – 10). Thus, FTR can be a potential option along with other ARVs to treat PWH who are failing regimens containing current ARVs. Our study shows potential for the use of FTR in this setting as we find similar prevalence of FTR resistance-associated polymorphisms in both ART naïve and individuals on ART with VF with no prior FTR exposure. We recommend further studies on the phenotypic impact of these polymorphisms are warranted to guide how to monitor for FTR resistance.

## SUPPLEMENTAL MATERIAL

Table S1 spectrum.01251-23-s0001.tif Prevalence of polymorphisms at the positions associated with fostemsavir resistance.

ASM does not own the copyrights to Supplemental Material that may be linked to, or accessed through, an article. The authors have granted ASM a non-exclusive, world-wide license to publish the Supplemental Material files. Please contact the corresponding author directly for reuse.
