# Supplementary figures and images for: Fostemsavir resistance-associated polymorphisms in HIV-1 subtype C in a large cohort of treatment-naïve and treatment-experienced individuals in Botswana
Source: Microbiol Spectr. 2023 Oct 12;11(6):e01251-23. doi: 10.1128/spectrum.01251-23 (PMC10714836; doi:10.1128/spectrum.01251-23)

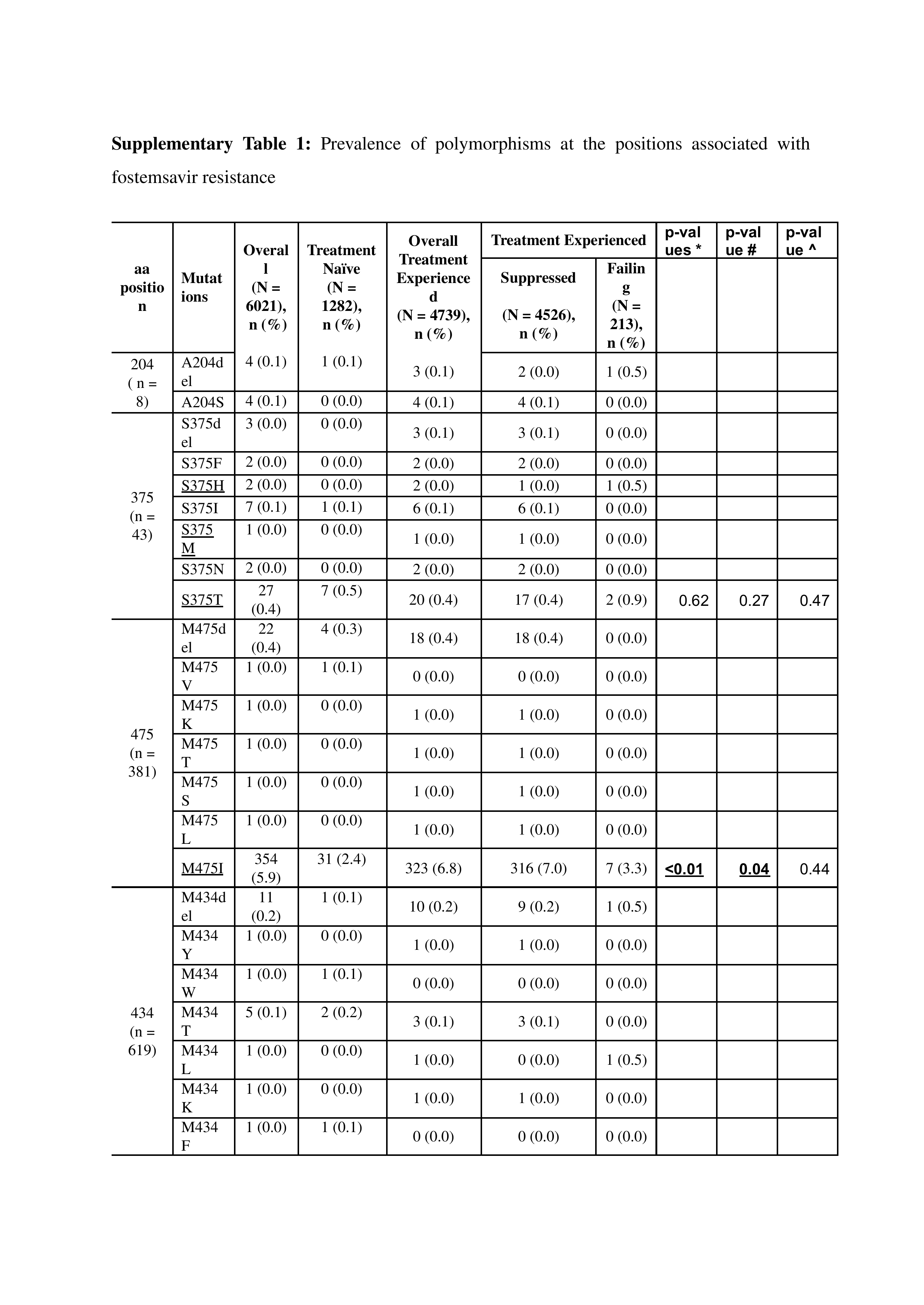

Supplement: Table S1 — Prevalence of polymorphisms at the positions associated with fostemsavir resistance. [file spectrum.01251-23-s0001.tif]
